# Edge-Enhanced Disruptive Camouflage Impairs Shape Discrimination

**DOI:** 10.1177/2041669519877435

**Published:** 2019-09-16

**Authors:** Rebecca J. Sharman, P. George Lovell

**Affiliations:** Division of Psychology, Abertay University, Dundee, UK

**Keywords:** camouflage, contours/surfaces, features/parts, object recognition, objects and features, shape, shapes/objects

## Abstract

Disruptive colouration (DC) is a form of camouflage comprised of areas of pigmentation across a target’s surface that form false edges, which are said to impede detection by disguising the outline of the target. In nature, many species with DC also exhibit edge enhancement (EE); light areas have lighter edges and dark areas have darker edges. EE DC has been shown to undermine not only localisation but also identification of targets, even when they are not hidden (Sharman, Moncrieff, & Lovell, 2018). We use a novel task, where participants judge which “snake” is more “wiggly,” to measure shape discrimination performance for three colourations (uniform, DC, and EE DC) and two backgrounds (leafy and uniform). We show that EE DC impairs shape discrimination even when targets are not hidden in a textured background. We suggest that this mechanism may contribute to misidentification of EE DC targets.

## Introduction

External disruptive colouration (DC) is a pattern comprised of contrasting coloured patches that create false edges that break up the outline of a target impeding localisation and identification (Cott, 1940; Cuthill et al., 2005; Egan, Sharman, Scott-Brown, & Lovell, 2016; Merilaita, Scott-Samuel, & Cuthill, 2017; Sharman et al., 2018; Thayer, 1909), whereas internal DC is concerned with perceptually breaking up the surface of the target using false correspondences and texture gradients (Merilaita et al., 2017). In this correspondence, the camouflage patterns investigated have features of both internal and external DC, thus “DC” is used as an inclusive term for both types of pattern. DC is often accompanied by edge enhancement (EE), where light patches have lighter edges and dark patches have darker edges (Egan et al., 2016; Osorio & Srinivasan, 1991; Sharman et al., 2018).

DC is particularly effective when combined with background matching, such that the coloured patches appear to belong to different background objects. This pattern results in differential blending of the contrasting patches (Cott, 1940), disrupting grouping and perceptual organisation mechanisms (Espinosa & Cuthill, 2014). In order for this to work, coloured patches must appear more different from each other than from adjacent background colours, causing them to be grouped with the background rather than with each other (Espinosa & Cuthill, 2014). In this fashion, DC disrupts higher level feature integration, specifically figure-ground segregation. However, edge-enhanced DC hinders identification of targets even when they are presented on a contrasting background (Sharman et al., 2018). This suggests that this form of camouflage may also disrupt lower level visual processes.

Some of the earliest research into edge-enhanced disruptive camouflage suggests that it works by super-exciting edge detectors, causing false edges to become more salient than real edges (Osorio & Srinivasan, 1991). This is supported by computational models that show DC disrupts detection of target outlines (Stevens & Cuthill, 2006). There are several suggestions as to how exactly this disruption may take place. It may be as simple as the false edges creating perceptual noise, decreasing the signal-to-noise ratio (Merilaita et al., 2017). However, it seems likely that it also involves some form of neuronal inhibition. Lateral inhibition or surround suppression would suggest that the high-contrast false edges reduce sensitivity to nearby lower contrast real edges, particularly those which are perpendicular to the false edges (Merilaita et al., 2017; Troscianko, Benton, Lovell, Tolhurst, & Pizlo, 2009).

If DC interferes with contour integration how might that work: What mechanisms might be involved? High-contrast contours, such as the false edges in DC, invoke strong end-inhibition, which may suppress perception of the actual outline of the animals (Field, Hayes, & Hess, 1993; Kapadia, Westheimer, & Gilbert, 1999). Embedding contours in a textured surround reduces neuronal firing, compared to the same length contour on a uniform background. However, embedding a high-contrast contour within a texture also changes the spatial summation properties of receptive fields, such that longer contours increase neuronal firing (Kapadia et al., 1999). This could mean that the long high-contrast contours of DC are enhanced, relative to the low-contrast contours of the true outline, when they are embedded in a cluttered (textured) natural environment. The neural representation of the false edges may be enhanced relative to the true edges of the animal.

Currently, the idea that DC disrupts contour integration and shape perception is largely theoretical. There is a lack of behavioural evidence that DC actually affects our ability to accurately perceive the outline of a target. We use a novel approach, spatial perturbation detection or “wiggle” detection, to address this question. “Snake” shapes are perturbed in space and subjects are asked to detect which of two shapes is more “wiggly,” that is, which has greater curvature. The paradigm aims to measure the point at which curvature differences are just noticeable; the detection threshold for “wiggle.” This technique investigates whether DC interferes with contour-shape perception. For example, if wiggle is harder to discriminate when stimuli have EE DC, compared to DC alone, this would suggest that EE directly affects shape perception.

## Method

All research materials are available on the Open Science Framework (Lovell & Sharman, 2019).

### Participants

A total of 26 observers (including author PGL) participated in the experiment. All participants had normal or corrected-to-normal vision. Observers gave their informed consent and were treated in accordance with the Declaration of Helsinki (2008, Version 6). Ethical approval for the study was granted by the Abertay University Social and Health Sciences Ethics Panel.

### Stimulus Generation

Stimuli were presented on a gamma-corrected 21-in. Sony Trinitron cathode ray tube (CRT) monitor (GDM-F520) with a spatial resolution of 1,280 × 1,024 pixels and a refresh rate of 60 Hz. All stimuli were presented in the centre of the monitor on a mid-grey background with average luminance of 65.3 cd/m^2^ and with colour 0.28, 0.30 in CIE xy coordinates, measured with a colourimeter (ColourCal Mk II, Cambridge Research Systems, Cambridge, UK). We used a chin-rest, which ensured a constant viewing distance of 60 cm. All stimuli were generated in Matlab (Version 8.4, The MathWorks Inc., Natick, MS, 2014), and all data were collected using PsychoPy (Peirce, 2007).

**Figure 1.**
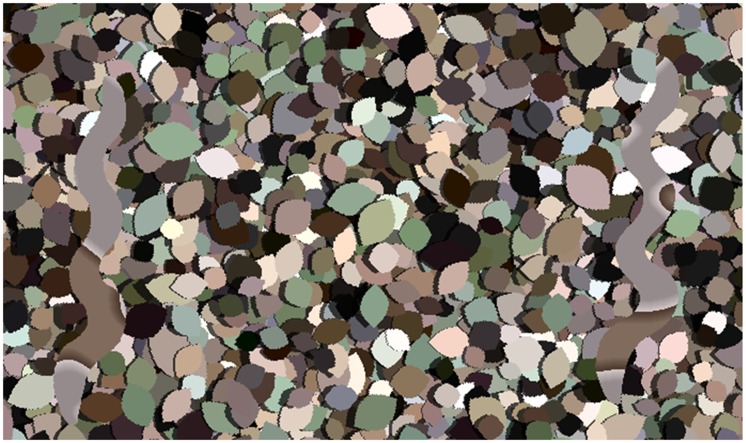
Example stimulus, showing two edge-enhanced target stimuli on the leaf background. In this instance, the snake shape on the right has a larger wiggle amplitude.

Stimuli featured two snake-shaped regions on either a mean-grey background or a background of leaf-shaped blocks of colour (Figure 1). The leaf backgrounds were generated in the same manner as described in Egan et al. (2016). Ten different random leaf backgrounds were generated, and each had 4,096 leaves (mean left height = 0.68°). A “shadow” was created leftwards of each leaf area by reducing the “L” channel by 30% in LAB luminance space (McLaren, 1976). The overall size of the stimulus was 1,024 × 1,024 pixels (corresponding to 29.86° visual angle).

The snake shapes were presented side-by-side, 15.9° from the centre (Figure 1). However, each had a random y-offset of between −1.49° and 1.49° in order to prevent participants from basing their judgements upon phase alignments between the snake images.

The “wiggle” in each stimulus was created by taking a bitmap image of a snake (the same clipart image used in Egan et al., 2016). The baseline amount of wiggle was based upon the distance between the leftmost and rightmost pixels within the snake image (82 pixels; corresponding to a visual angle of 2.44°). All snakes were 9.73° from top to bottom. Manipulations of the amount of wiggle were achieved by resizing the width of the bitmap image. An amplitude of 0.5 would halve baseline width, while an amplitude of 2 would double the baseline width. The baseline and width-manipulated snakes were randomly assigned to left and right sides of the stimulus on each trial. Camouflage textures of the same dimensions as the leaf background were created and then sampled using the snake-shaped masks. The variability of the y-offset, randomisation of position (left/right), and variations in the wiggle amplitude mean that no two samples would be precisely the same.

**Figure 2.**
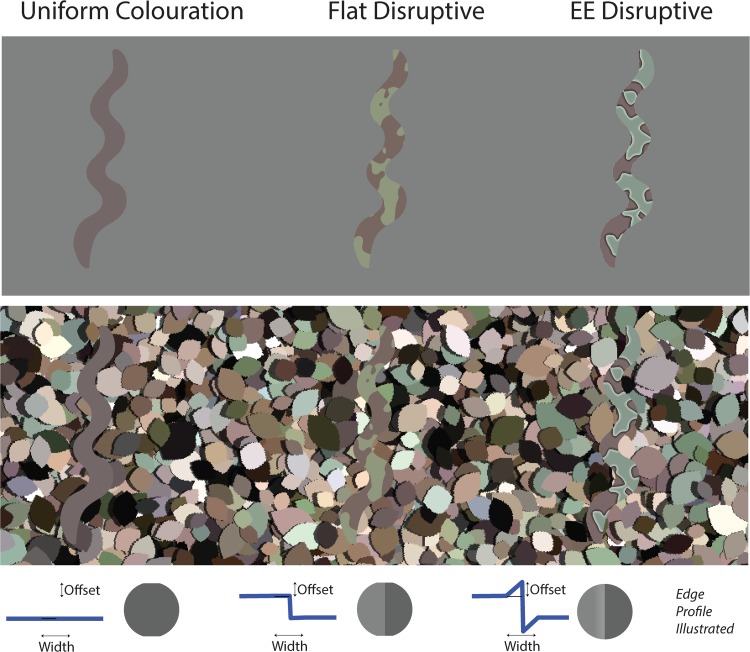
Illustration of the three stimulus conditions: uniform, flat disruptive, and edge-enhanced disruptive on both uniform and leaf backgrounds. The lower row shows the luminance profile of the colouration. The offset is the change in luminance (CIE L) added to light patches or subtracted from dark patches. The width is the spatial range over which this luminance range occurs. (Top) Target stimuli against a mean grey background, (middle) the same target stimuli against a “leaf” background, and (bottom) an illustration of the luminance profile across camouflage patch edges. EE = edge enhancement.

There were two types of camouflage (flat disruptive and edge-enhanced disruptive) and a uniform coloured control (Figure 2). The camouflage patterns were generated in the manner described in Egan et al. (2016) and Sharman et al. (2018). The patterns were created by first generating a white noise image. This was then filtered in the Fourier domain (Equation (1)), where *d* is the distance from the centre of the image (in Fourier space), μ was 0.07, and σ was 120.

(1) $$\text{Filter}=e^{\frac{-{\left(d-\text{μ}\right)}^{2}}{2{\left(\text{σμ}\right)}^{2}}}$$

**Figure 3.**
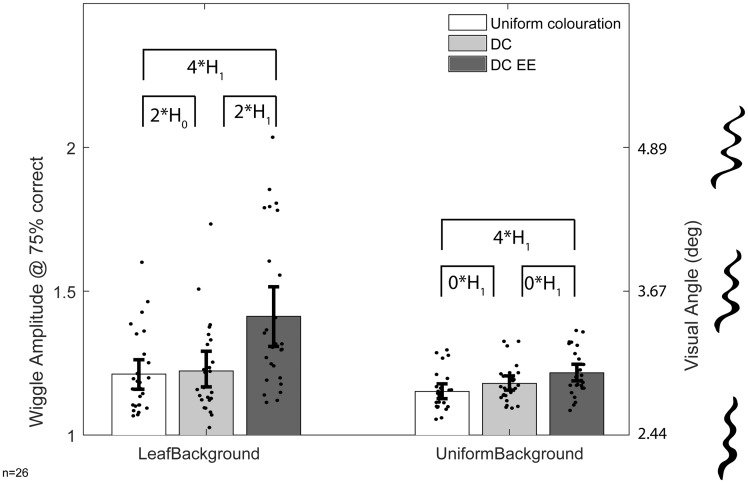
Experimental results. The thresholds for the conditions on the leaf background are shown on the left and those for the uniform background are on the right. The thresholds for the uniform condition are shown in white, DC in mid-grey, and EE DC in dark grey. Amplitude is shown as multiples of baseline stimulus width. Error bars represent the bootstrapped 95% confidence intervals; dots are jittered raw data. Comparisons show a summary of the Bayesian *t* test results: 0* = *anecdotal*, 1* = *moderate*, 2* = *strong*, 3* = *very strong*, and 4* = *extreme evidence* for H0 or H1 (the null and experimental hypotheses, respectively). DC = disruptive colouration; EE = edge enhancement.

The filtered texture was then posterised by assigning different colour values to the values above and below the 50th percentile. These colours were chosen from the same sample as that used for the leaves. Within that population, one colour was chosen from those pixels with a brightness level within the 35th to 45th percentiles and the other from the 55th to 65th percentiles.

The EE was created in the manner described in Egan et al. (2016) and was defined by two parameters: the total width, the spatial extent of the EE including both sides of the pattern element boundaries, was 0.63°, and the offset, difference in brightness from the pattern element towards the enhanced edge was 60 (in CIE L units).

Ten textures were generated for each camouflage type, and one was randomly selected for each trial; in each trial, one of the leaf backgrounds was randomly selected along with one of the available random camouflage patterns.

### Procedure

A two-alternative forced choice (2AFC) design was employed to measure the point at which the difference in wiggle amplitude was just noticeable: the wiggle discrimination threshold. We used a pedestal technique, where in each trial one of the snakes (the foil) had a fixed wiggle amplitude of 1.0 and the other (the target) had a wiggle amplitude varied in accordance with a staircase procedure. On each experimental trial, we added an offset to the degree of *wiggle* to both stimuli, this was randomly selected value between −.2 and +.2 in steps of 0.05. This prevented participants from basing their responses on the baseline stimulus without examining the manipulated shape. Each stimulus was presented until participants made a response, with a minimum presentation time of 1,000 ms. There was an interstimulus interval of 2,000 ms. The observers’ task was to indicate, by key press, which snake had the highest wiggle amplitude; which was the most “wiggly.”

Two staircases were used for each experimental condition (camouflage type and background type): one starting with an amplitude of 1.2× baseline width and the other starting with 0.8× baseline width. The staircases were designed to converge at the 79.37% threshold and were terminated after 30 trials (360 trials total across conditions for each participant). All conditions were run within the same block, with each trial being randomly selected from the available staircases.

## Results

All data (raw and summarised) and analysis scripts are available on the Open Science Framework (Lovell & Sharman, 2019).

Each set of 60 trials per participant, per condition, was fit with a logistic function (ɣ = 0.5, λ = 0, α and β were determined using a least squares fit). The wiggle discrimination threshold was determined as the point at which the logistic value passed through 75% correct (Figure 3).

We use a Bayesian approach to data analysis because it offers robust and transparent inferences. Bayesian analyses compare the probabilities for both the null and experimental hypotheses, thereby reducing the likelihood of a Type-I error (Dienes, 2011). We classify the magnitude of Bayes factors into different evidential strengths (*extreme, very strong, strong, moderate*, and *anectodal*) based on Jeffreys (1961) and Lee and Wagenmakers (2013).

We conducted a two-way Bayesian analysis of variance, using JASP (Love et al., 2015), with factors background (uniform vs. leaf) and colouration (uniform vs. flat disruptive vs. edge-enhanced disruptive) and wiggle threshold as the dependent variable. The preferred model, with the largest Bayes factor (BF10 = 4.476 × 10^8^, *extreme evidence*, BF > 100) included all main effects and interactions. We then conducted within subjects Bayesian *t* tests (JASP, Love et al., 2015) to explore which wiggle thresholds were different from one another (for details of nonsignificant differences, see Lovell & Sharman, 2019). When presented upon a leaf background, the EE DC threshold was different from flat DC threshold (BF = 21.375, *strong evidence*, BF > 10) and from uniform colouration (BF = 86.509, *extreme evidence*). Against the uniform grey background, there was extreme evidence for a difference in wiggle thresholds between EE DC and uniform colouration (BF = 181.107, *extreme evidence*).

Finally, we conducted Bayesian one-sample *t* tests comparing wiggle thresholds to the baseline level of wiggle (1.0). We find extreme evidence for all thresholds being greater than baseline. This confirms that participants were able to complete the task under all conditions.

## Discussion

Edge-enhanced disruptive colouration (EE DC) increases wiggle discrimination thresholds; participants were worse at judging the shape of targets with EE DC compared to DC and uniform targets on a leaf background. Thresholds are also increased for EE DC compared to uniform targets even when clearly visible on a simple uniform grey background.

These findings present the intriguing possibility that EE DC is not only making target outlines harder to see via differential blending (Espinosa & Cuthill, 2014), but it is also causing a misperception of the amount of curvature and therefore also of the location of the contours. Potentially, causing nonveridical perception of an object’s objects and, therefore, increasing the difficulty of identifying objects.

Previous research has shown, via a computational model of edge detection, that DC disrupts the *detection* of body outline. Our results suggest that *identification* is also affected; therefore, DC may not only disrupt figure-ground segregation but also shape perception. This adds to an increasing body of work looking at perceptual effects of camouflage beyond concealment. For example, dazzle camouflage (highly conspicuous and often repetitive patterns) has been shown to affect speed perception (Scott-Samuel, Baddeley, Palmer, & Cuthill, 2011) and target tracking (Hogan, Cuthill, & Scott-Samuel, 2016).

We propose that the disruption of target recognition occurs via a mechanism, whereby edge signals are minimised and false-edge noise is increased; changing the signal-to-noise ratio (Merilaita et al., 2017). Our stimuli may show this effect particularly clearly, due to the properties of curved stimuli. Curved segments are more important than straight segments for identifying simple outlines (Panis & Wagemans, 2009). Global shapes can be represented by their maximum convex, maximum concave, and inflection points (Bell, Hancock, Kingdom, & Peirce, 2010). Thus, our stimuli create a strong neural representation. This is also appropriate to camouflage research, in particular, as animals have generally more curved configurations than man-made structures (Perrinet & Bednar, 2015).

However, there is also evidence that curvature reduces salience. Snake-like paths are harder to detect even when local curvature is identical (Loffler, 2008). It has been suggested that this is because the sudden changes in curvature weaken the association fields (Field et al., 1993) in that area (Pettet, McKee, & Grzywacz, 1998). Therefore, the shape of the stimulus may be further enhancing the effect of DC, by disrupting the association fields between the neurons representing the target outline.

The current results support existing evidence that EE DC affects not only target detection but also identification mechanisms (Sharman et al., 2018). We have shown that this form of colouration undermines object recognition mechanisms, potentially by undermining curvature discrimination and subsequently shape discrimination. This effect occurs even when the target is readily visible, again underlining the utility of EE DC not only in concealing targets but also in disguising their identity. This complements existing evidence that shape is a principal discriminative cue in animal detection in natural scenes (Elder & Velisavljevic, 2009).
